# Direct comparison of in-vivo and post-mortem spin-echo based diffusion tensor imaging in the porcine heart

**DOI:** 10.1186/1532-429X-17-S1-P76

**Published:** 2015-02-03

**Authors:** Christian T Stoeck, Constantin von Deuster, Nikola Cesarovic, Martin Genet, Maximilian Y Emmert, Sebastian Kozerke

**Affiliations:** 1 Institute for Biomedical Engineering, University and ETH Zurich, Zurich, Switzerland; 2 Imaging Sciences and Biomedical Engineering, King's College London, London, UK; 3 Department of Surgical Research, University Hospital Zurich, Zurich, Switzerland; 4 Clinic for Cardiovascular Surgery, University Hospital Zurich, Zurich, Switzerland

## Background

Spin-echo based cardiac diffusion tensor imaging (DTI) is highly sensitive to myocardial strain [1]. Imaging during systolic contraction requires precise planning of the sequence timing [2]. Second order motion compensated diffusion encoding has recently been proposed for small animal imaging [3] to reduce the impact of myocardial strain on the diffusion tensor.

It is the objective of the present work to compare second order motion compensated spin-echo DTI of the in-vivo and post-mortem porcine heart on a clinical MR system.

## Methods

**Figure 1:**
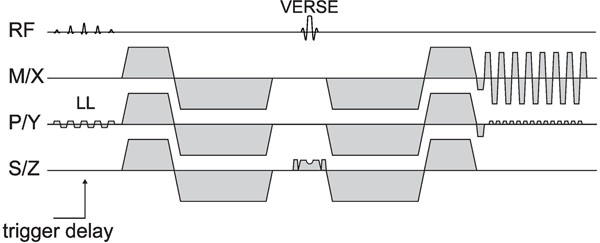
**Sequence diagram of the second order motion compensated diffusion sequence.** A spatial spectral reduced field of view (LL) excitation is used for fat suppression. A variable rate selective excitation (VERSE) echo pulse is used for refocussing.

**Figure 2:**
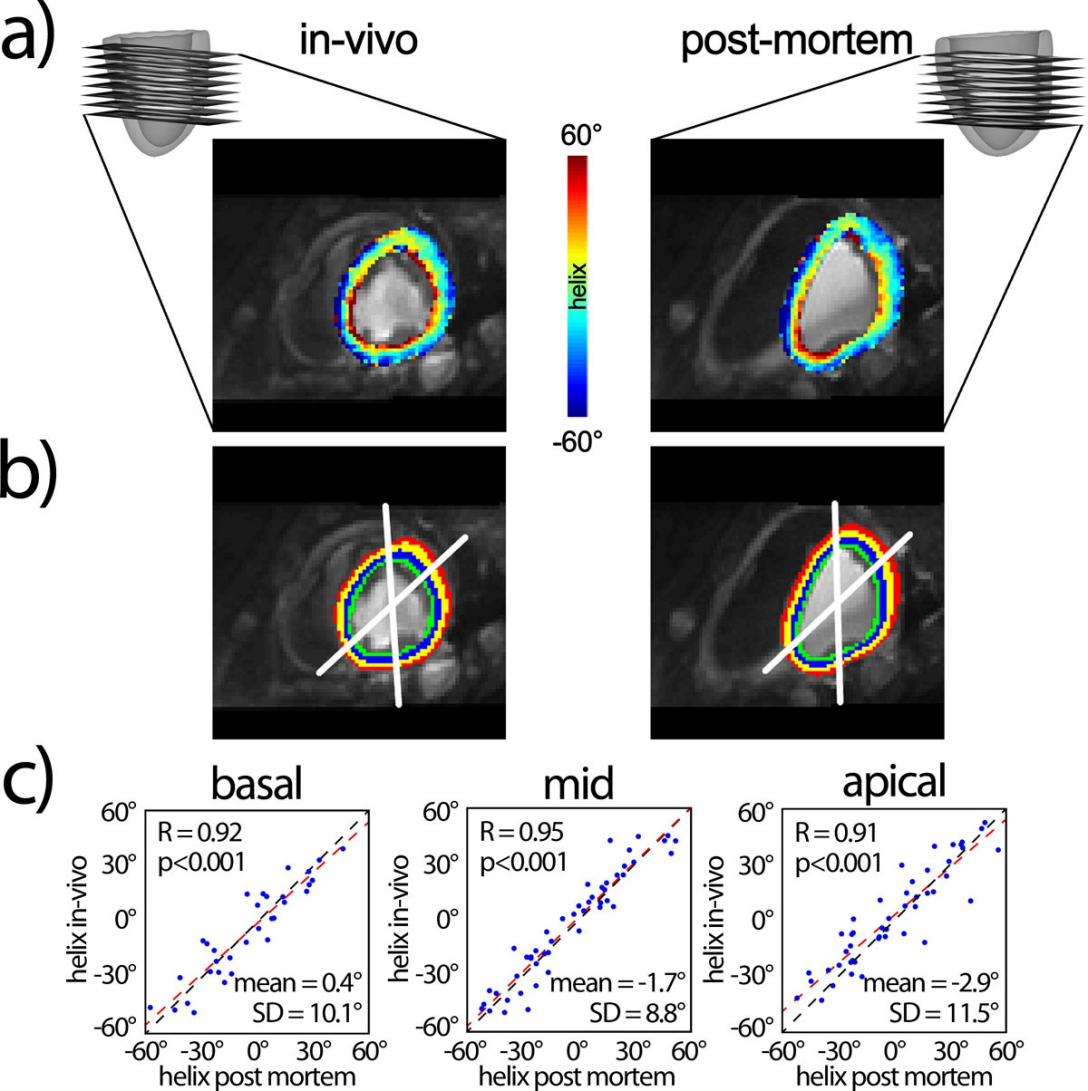
**Slice distribution and example helix angle maps for in-vivo and post-mortem imaging are shown in a). Four angular and four radial sectors b) were identified.** Mean helix angles per sector were spatially matched and correlation analysis is shown in c). The mean and one standard deviation (SD) of the differences between in-vivo and post mortem are reported.

Second order motion compensated diffusion encoding gradients were incorporated into a cardiac triggered single-shot spin-echo sequence (Figure 1). A pig (55kg) was imaged on a 1.5T clinical system (Philips Healthcare, Best, The Netherlands) equipped with a gradient system delivering 80mT/m@100mT/m/ms. Eight slices (Figure 2a) were acquired during free breathing with the following parameters: resolution: 2.2×2.2mm^2^, slice thickness: 6mm, reduced FOV [4]: 230×98mm^2^, TR/TE: 2R-R/83ms, 10 averages. Fat suppression was established by spectral-spatial excitation. Ten diffusion-encoding directions [5] with a b-value of 450s/mm^2^ were applied during early systole. The pig was euthanized by a potassium injection inside the MR scanner and the imaging protocol was repeated. Helix angle maps were calculated upon tensor reconstruction [6]. The myocardium was segmented in 4 angular and 4 radial segments per slice similar to the procedure proposed in [7] (Figure 2b).

## Results

Example helix angle maps are shown in Figure 2a). Sectors were spatially matched and the corresponding correlation analysis for in-vivo vs. post-mortem data are presented in Figure 2c). Root mean squared differences between sectors in-vivo and post-mortem were 10.0°, 8.9° and 11.7° for basal, mid and apical levels, respectively. Despite significant deformation of the post-mortem heart due to the loss in blood pressure, good agreement between in-vivo and post-mortem data is revealed.

## Conclusions

Good correlation between in-vivo and post-mortem imaging was found proving that bulk motion and strain effects are well suppressed if second order motion compensated diffusion gradients are employed.

## Funding

EU FP7 Marie-Curie fellowship to MG.
